# “Community 101 for researchers”: an online training program to build capacity for ethical community-engaged research with Native Hawaiians and Pacific Islanders

**DOI:** 10.3389/fpubh.2023.1121748

**Published:** 2024-01-05

**Authors:** Jane J. Chung-Do, Samantha Keaulana Scott, Bradley R. Jones, Mele A. Look, Deborah A. Taira, Neal A. Palafox, Kamahanahokulani Farrar, Marjorie K. Leimomi M. Mau

**Affiliations:** ^1^Office of Public Health Studies, Thompson School of Social Work and Public Health, University of Hawai‘i at Mānoa, Honolulu, HI, United States; ^2^Pacific Biosciences Research Center, School of Ocean and Earth Science and Technology, University of Hawai‘i at Mānoa, Honolulu, HI, United States; ^3^Department of Native Hawaiian Health, John A. Burns School of Medicine, University of Hawai‘i at Mānoa, Honolulu, HI, United States; ^4^Daniel K. Inouye College of Pharmacy, University of Hawai‘i at Hilo, Hilo, HI, United States; ^5^Department of Family Medicine and Community Health, John A. Burns School of Medicine, University of Hawai‘i, Honolulu, HI, United States; ^6^Papa Ola Lōkahi, Honolulu, HI, United States

**Keywords:** community-engaged research, participatory research, research ethics, training, Native Hawaiians, Pacific Islanders, Hawaii

## Abstract

To address the history of unethical research and community distrust in research among Native Hawaiian and Pacific Islander communities, we developed the “Community 101 for Researchers” training program, which was launched in 2014 to enhance the capacity of researchers to engage in ethical community-engaged research. The purpose of this paper is to describe the development of this training program as well as its reach and feedback from participants. The Community 101 training program is a self-paced, 2-h online training program featuring community-engaged researchers from the University of Hawai‘i and their longstanding community partners. Throughout the five modules, we highlight the historical context of Native Hawaiians and Pacific Islander populations in Hawai‘i related to research ethics and use examples from the community as well as our own research projects that integrate community ethics, relevance, benefits, and input. To determine reach and gather participant feedback on the training, we extracted data from the user accounts. The training has been completed by 697 users to-date since its launch. Despite very little advertisement, an average of nearly 70 users have completed the Community 101 Program each year. The majority of the participants were located in Hawai‘i though participants were also from other states and territories in the US, and international locations. The majority of participants were from universities in Hawai‘i in 51 different departments demonstrating multidisciplinary relevance of the program's training. The general feedback from the 96 participants who completed an optional anonymous evaluation survey given at the end of the training was positive. The “Community 101 for Researchers” Training program is an accessible and relevant tool that can be used to advance ethical community engaged research, specifically with Native Hawaiian and Pacific Islander communities.

## 1 Introduction

Similar to other historically marginalized communities, Native Hawaiian and Pacific Islander communities face pervasive and unjust health disparities. Although health research has attempted to address these disparities, communities are frustrated by the legacy of unethical research practices that fail to meaningfully involve them. Thus, the “Community 101 for Researchers” training program was launched in 2014 to enhance the capacity of researchers to engage in ethical community-engaged research with Native Hawaiians and Pacific Islanders, particularly in Hawai‘i. The purpose of this paper is to describe the development of “Community 101 for Researchers” training program to promote ethical research with these communities as well as the reach of the training program and feedback from participants.

Native Hawaiians and other Pacific Islanders are culturally rich communities that share common values and practices but also have unique and distinct cultures. Native Hawaiians are the Indigenous people of the Hawaiian Islands. They arrived in the Hawaiian Archipelago by wa'a (canoe) from the Marquesas Islands 1,600 years ago. Other Pacific Islanders have migrated or immigrated to the Hawaiian Islands and represent a culturally diverse range of communities across the Pacific, including Samoans, Tongans, Guamanians/Chamorro, Micronesians, and Fijians. Native Hawaiians and Pacific Islanders were healthy and robust people who developed sophisticated agroecological food systems with spiritual ties to the land and surrounding nature and strong familial and collectivistic values that sustained their communities. With colonization and militarization came the decimation of the populations by introduced infectious diseases, development of land for capitalistic and militaristic gains, and marginalization from socioeconomic opportunities. Although the State of Hawai‘i is often portrayed as home to the healthiest populations in the US (1), pervasive and unjust health disparities exist among Native Hawaiians, Pacific Islanders and other marginalized populations in the state. For example, Native Hawaiians and Pacific Islanders have lower life expectancy and die at higher rates from coronary heart disease, stroke, congestive heart failure, cancer, and diabetes than other residents in the state of Hawai‘i (2–5). Native Hawaiians are less likely to live in neighborhoods with healthy food grocers (6, 7) and have less economic means to buy healthy food. With Hawai‘i being one of the most expensive places to live in the US (8), Native Hawaiians have a more difficult time paying for essentials, such as housing, utility bills, medicine, child care, and food compared to the average resident in Hawai‘i. In fact, 34% of Native Hawaiians report facing financial challenges purchasing food (9). With a total of 14.8% of Native Hawaiians and Pacific Islanders living in poverty compared to 9% of non-Hispanic white individuals (10), it is becoming increasingly impossible for many Native Hawaiians to live in their ancestral homes. Hawai‘i has the highest rate per capita of homelessness with more than 50% of all the houseless individuals identifying as Native Hawaiian and/or other Pacific Islander (11). Given these structural barriers, it is not surprising that Native Hawaiians and Pacific Islanders are more likely to deal with depression, anxiety, substance use, and suicidal behaviors (3, 12).

Despite these disparities and forces of oppression, Native Hawaiians and Pacific Islanders have survived and thrived. Since the 1970's, the Native Hawaiian Renaissance Movement has made tremendous efforts and strides in revitalizing Native Hawaiian cultural practices, such as the Hawaiian language, oceanic voyaging, food cultivation, and land and ocean restoration (13–15). Pacific Islander communities who now call Hawai‘i their home have also retained their cultural practices and have come together to take care of their families and communities. Composing only 4% of Hawai‘i's total population, the Pacific Islander populations disproportionately comprised 24% of the COVID-19 infections from March 2020 through January 2021 during first 9 months of the pandemic in Hawai‘i (16). Because the State's COVID-19 response to Pacific Islanders was severely insufficient, Pacific Islander community leaders proactively took matters into their own hands to protect their communities. They organized assistance with filing for unemployment and housing relief, conducted outreach concerning prevention efforts, developed messaging relevant to the need for physical distancing, fundraising for personal protection equipment, funeral costs, and groceries, and provided language assistance and translated materials to local organizations serving Pacific Islanders (17). Thus, cultivating research partnerships that engage community leaders and recognize the strengths and assets of the community may help restore community wellness (2, 18–21).

Although notable strides have been made in Native Hawaiian and Pacific Islander research, researchers have inflicted harm by conducting helicopter research, defined as “any investigation within the community in which a researcher collects data, leaves to disseminate it, and never again has contact with the tribe” (22). Well-intentioned researchers who are often outsiders of the “target” community perpetuate this type of research because they lack understanding of the community and its historical and cultural background, which has led to exploitation and ethical breaches (23, 24). Most of these “target” communities face racism, oppression, and social injustices and have vastly different lived realities from the outside researchers (25). Like other minoritized communities, researchers have continued to inflict harm on Native Hawaiian and Pacific Islanders through a long history of exploitative and unethical research studies. For example, the US government conducted 12 years of nuclear weapons testing on inhabited atolls in the Pacific Ocean, knowingly exposing many Pacific Islanders to harmful radiation. The nuclear testing is associated with significant increases in the incidence and prevalence of radiogenic cancers in these populations (26). In the 1860's, Hansen's Disease, which was once known as leprosy, spread throughout the Hawaiian Islands. As with other infectious diseases, Native Hawaiians were disproportionately infected with Hansen's disease. Despite very little understanding of disease transmission, infected people were forcibly taken from their families and exiled to a remote northern peninsula on the island of Moloka‘i as a public health strategy. In addition to the limited resources and infrastructure available to those who were exiled, unethical medical studies were conducted by government physicians to identify the mode of disease transmission without consent or regard for this vulnerable population (27). Community members continue to express their frustration with researchers who are disrespectful of their cultural protocols, hold damaging stereotypical notions of their community, and provide no perceivable benefits to the community (28). This legacy of exploitative and extractive research has rightfully led to the distrust and suspicion of researchers. Some of these challenges have incrementally improved largely due to the growing number of research scientists who are trained in participatory research and decolonizing methodologies with many who are ancestrally tied to these communities. However, there remains ongoing examples of well-intentioned but unethical research approaches from other researchers (28). Thus, methods to increase awareness and capacity among all researchers who wish to work ethically and meaningfully with Native Hawaiian and Pacific Islander communities are critically needed. From the Native Hawaiian and Pacific Islander context, ethical research means centering all engagement and decisions in cultivating relationships, demonstrating long-term commitment beyond grant timelines, and taking the time to learn and acknowledge the uniqueness of each community to promote cultural safety (27, 28). Ethical research also means to ensure the community truly benefits from the research activities by engaging community members and leaders throughout the stages of the research process.

To address the history of unethical research and community distrust in research, researchers have developed educational tools to increase researchers' awareness and capacity to ethically and effectively engage with communities. Researchers have developed various training programs, such as the Collaborative Institutional Training Initiative (CITI Program), which is required by many Institutional Review Boards (29). The National Institute of Health (NIH) also offers the “Introduction to the Responsible Conduct of Research” online training (30). In an effort to increase the number of researchers who are able to identify and address “the ethical, legal, and social implications of their research,” the NIH also solicited training grant proposals from 1999 to 2004 (31). Through 2007, the NIH T15 “Short-Term Courses in Research Ethics” program supported a total of 26 different training programs with each program focusing on a specific type of research (e.g., behavioral, clinical, or genetic) or a specific population of research participant (e.g., international, minority, or vulnerable participants) (32). Other trainings have been developed by universities and organizations to address these needs (33, 34), with some of these educational tools being in written manual form and others designed as online training programs (34). However, research ethics training and university ethics review boards have been long criticized for applying an individualistic westernized perspective on research ethics, which fails to take community-level impact and harm into account (35). Therefore, there has been a growing effort to integrate guidelines and principles of community-engaged research and decolonizing methodologies into these research ethics training tools that are culturally tailored for specific communities. However, a limited number of tools exist that are specifically tailored for researchers who are interested in collaborating with Native Hawaiian and Pacific Islander populations.

In response to this gap, we developed and launched the “Community 101” training program in 2014. We are a multidisciplinary group of both “insider” and “outsider” community-engaged researchers who have expertise in medicine, public health, Native Hawaiian health studies, business management, and economics. We each have 10–30 years of experience conducting community-engaged and health disparities research that have been funded by local and national agencies including the National Institutes of Health, Department of Health and Human Services, American Diabetes Association, Robert Wood Johnson Foundation, Hawai‘i Community Foundation. The Community 101 program was a joint project of the RCMI Multidisciplinary and Translational Research Infrastructure Expansion (RMATRIX) and the Center for Native and Pacific Health Disparities (CNPHDR) at the University of Hawai‘i John A. Burns School of Medicine funded by the National Institute of Health (36). The purpose of the Community 101 Training was to provide a training tool for new investigators who are interested in working with Native Hawaiian and Pacific Islander communities. The training was named “Community 101” because it was initially a part of a training series within the Community Engagement division of the CNPHDR. The series provided community health allies, such as community health workers, with chronic disease training, which were named Diabetes 101, Kidney 201, Heart 101, etc. The title was also given to convey the message that this is an introductory training and more learning is needed to establish advance competence.

Together, we developed a 2-h self-paced online training composed of five modules that highlight the historical context of Native Hawaiians and Pacific Islander populations in Hawai‘i related to research ethics and use examples from the community as well as our own research projects that integrate community ethics, relevance, benefits, and input. The purpose of this paper is to describe the development of the Community 101 online training program as well as its reach and feedback from participants.

## 2 Pedagogical framework

### 2.1 Development of the Community 101 Training Program

The Community 101 Training Program was developed and launched in 2014 through a partnership between academic faculty and community-based organizations who had been working together on clinical care programs, research studies and/or public health activities serving Native Hawaiian and Pacific Islander populations throughout the state. The longstanding relationships with community-based organizations using culturally appropriate engagement approaches, bi-directional learning and equitable sharing of resources (i.e., funding, etc.) allowed for open discussions about the need to educate future academic researchers on lessons learned about building durable and trustworthy relationships with community-based organizations throughout the state and in particular, focusing on community organizations serving primarily Native Hawaiian and Pacific Islander populations. One of the authors (MKM) conceived of the idea to create an online program similar to a “National Cancer Institute-like” online research ethics training program. The concept was to teach participants about Human Subjects Protection for NIH-funded research and to adapt the NIH-model into a 5-module online program that would educate researchers about conducting community engaged research with Native Hawaiian and Pacific Islander populations. A diverse team of community-engaged researchers was assembled who approached select community organizations to participate in the establishment of the online program. It would provide an introductory course on “Research with Native Hawaiian and Pacific Islander communities” designed specifically for researchers with the intent of learning more about community-engaged research approaches. We each had multiple conversations with our community partners from Native Hawaiian-serving organizations including Kula no nā Po‘e Hawai‘i, God's Country Waimānalo, Wai‘anae Coast Comprehensive Health Center, etc to identify key areas of knowledge and skills researchers should be aware of and develop when working with communities. They recommended that we highlight the importance of establishing relationships, cultivating love and respect for the community, and data disaggregation and sharing data ownership. Therefore, one of the modules includes a video of community partners sharing their experiences, lessons learned, and advice for researchers to promote community voices in this training. In addition, key informant interviews with 24 community organizations across the Hawaiian Islands were analyzed to also inform the contents of the modules (27). We met over several months to discuss the ideas and themes that emerged from these multiple sources. We shared the draft of the training with our community partners and key informants to refine the content. The resulting five modules were finalized, which focused on:

- The historical and cultural context of Native Hawaiian and Pacific Islander populations in Hawai‘i and the US and how historical events has impacted their health and wellness.
- How Native Hawaiian and Pacific Islanders have been harmed through research and current considerations about research ethics and policies.
- How one's “biases” and background as a researcher may impact the way in which one approaches and works with Native Hawaiian and Pacific Islander communities.
- Determining the relationship expectations, priorities, and preferences of community organization and researchers serving Native Hawaiian and Pacific Islander populations.
- Defining community-based participatory research and how it may be implemented with communities in a meaningful way to engage them in research aimed at eliminating health inequities.
- Successful models for engaging communities in the research enterprise by examining real-life examples of NIH-funded community-engaged research projects with Native Hawaiian and Pacific Islander-serving community organizations.

We created the modules using a commercial product (Articulate) that combined an audio voice track with a PowerPoint presentation to create a self-contained HTML5 delivery mechanism. We also incorporated video clips were into some of the modules. In total, the five modules take about an average of 2 h to complete and are implemented as a Wordpress website. We each created post-module quiz questions that participants completed to help them measure their understanding of the module content. Participants must receive a minimum of 70% correct on the quiz to receive an online certificate. An optional evaluation survey is also available, which was developed by the researcher team. The survey includes the following six close-ended standard questions to evaluate their perception of the training program using a five point Likert scale from strongly disagree to strongly agree.

1. The overall teaching effectiveness of the instructors was excellent.
2. The overall quality of the modules was excellent.
3. The presentations were clear and easy to understand.
4. I learned a great deal from these presentations.
5. I feel well-prepared to interact with communities in developing research projects.
6. The software program used to present the modules was excellent.

In addition, participants were asked three open-ended questions asking about the strengths of the training and suggestions to improve the curriculum. Introductory pages are publicly available, but the training modules, quizzes and evaluation survey require a registered user to login. Wordpress plugins are employed to allow new users to self-register and another plugin allows registered users to access certain pages on the site. Registered users login to the website and study the modules at their own pace. The eventual program was launched with program funding from a second NIH-funded grant (RMATRIX) to record and edit the modules, upload and maintain the program platform, and manage data collection over the subsequent years.

### 2.2 Data extraction

To measure the reach of the Community 101 Program, we extracted self-reported data from the registered accounts of all users. To register, users were given open-ended optional questions that asked for their mailing address, department, organization, and their reason for taking the training. User data was extracted from a web page on the backend of Wordpress and imported into Excel. To examine usage over time, we extracted the year of when the quiz was completed for each user. Quiz data were exported from Wordpress in CSV (Comma Separated Values) format. To gather participant feedback on the curriculum, we extracted quantitative and qualitative data from the close-ended and open-ended questions from the optional survey that was provided at the end of the training program. Evaluation data for each respondent were copied manually from individual responses into an Excel spreadsheet.

### 2.3 Data analysis

We identified duplicate participants and only the first entry was kept and the remaining entries eliminated from the data set. To determine the reach, we examined the information provided by participants on their user account for “department” and “organization.” If these variables were not provided, we examined the mailing address if provided. Using this information, we categorized participant demographics into three categories. The first category was based on the geographic location of their organization. Any organization located in Hawai‘i was categorized as “Hawai‘i” We classified organizations located in other states and territories in the US as “other US states or territories.” Organizations located in countries outside of the US were designated as “international.” The second category was based on the type of department they were from. Departments mentioned by more than 10 users were identified. Departments with <10 users were collapsed into an “other departments” category. Organizations were coded into the most common organizational types, which included University, Hospital/healthcare, Government agency, Non-profit healthcare, Non-profit organization, Community, Business, and Military. Reason for completing the training program was qualitatively analyzed using content analysis.

To determine the number of users per year, we examined the dates of when the user completed the quiz. Because some participants completed the quiz more than once, only the first complete quiz results was recorded for that individual and the remaining “re-takes” were deleted. Participants who returned to the website to complete the program in a subsequent year(s) were notated as a “Multi-year User” and reflected in both years.

Responses on an optional evaluation survey measuring their perception of the training program consisting of six close ended questions using a Likert Scale (1 = strongly disagree to 5 = strongly agree). Responses to the evaluation survey were analyzed using a correlation matrix of items based on the four themes as follows: Theme 1 was designated as “Teaching Method” and included the first two questions; Theme 2 was noted as “Learner Impact” and included the third and fourth questions; Theme 3 was named “Learner Readiness” and included the fifth question and the 4th theme was named “On-line Teaching Platform” and included the sixth question. Each participant's response for each evaluation item was graphed on a 2-item matrix and grouped according to number of responses on both items and then categorized into High (Strongly Agree); Moderate (Agree) and Low (Neutral to Strongly disagree) perceptions for each specific theme. For the open-ended questions on the optional evaluation survey, two researchers coded the qualitative answers using content analysis to identify prevalent themes related to the strengths and areas of improvement for the program using the following steps. First, the researchers separately reviewed the participants' responses to the three open-ended questions and independently developed codes based on specific examples that were mentioned three or more times by at least three participants. Second, they met to review codes together, agreed upon a codebook, and recoded the answers independently. With consultation from the larger research team, they decided to categorize the responses into overarching themes of strengths or areas of improvement. Third, they met to compare the coded responses and determine saturation. Lastly, they met with the larger research team to discuss any disagreements and ambiguities to reach consensus on sub-themes.

## 3 Results

### 3.1 Participant characteristics

In total, the training was completed by 697 users in the period between February 2014 and Nov 2022 (see Table 1). The majority of the participants were located in Hawai‘i (67%), while a smaller number were from the continental United States and its territories including Puerto Rico (7%), and other countries (2%). The various states and US territories represented were California, Connecticut, Delaware, Georgia, Louisiana, Maryland, Michigan, Minnesota, Montana, New York, and South Carolina as well as Puerto Rico, Guam, and Saipan. There were participants from countries outside of the US, such as the United Kingdom, Canada, and Australia.

**Table 1:** Characteristics of participants who completed the “community 101 training for researchers program” (*n* = 697, 2014–2022).

**Characteristic**	***n* (%)**
**Geographic location of user**	
Hawai‘i	467 (67.0%)
Other US states or territories	50 (7.2%)
International (outside of USA)	13 (1.8%)
No response	167 (24.0%)
**Organization type**	
University	486 (69.8%)
Non-profit healthcare/organization	10 (1.4%)
Government agency, community, businesses, military	10 (1.4%)
Hospital/healthcare	9 (1.3%)
No response	182 (26.1%)
**Department affiliation**	
Public health	198 (28.4%)
Tropical medicine, medical microbiology, and pharmacology	55 (7.9%)
Native Hawaiian health	35 (5.0%)
Social work	21 (3.0%)
Pharmacy	16 (2.3%)
Nursing	15 (2.2%)
Cancer research	14 (2.0%)
Medicine	13 (1.9%)
Psychology	13 (1.9%)
Other	42 (6.0%)
No response	275 (39.4%)

The majority of participants were from universities and organizations in Hawai‘i such as the University of Hawai‘i, Chaminade University, Queen's Medical Center (see Table 1). Participants affiliated with academic and research institutions outside of Hawai‘i included Johns Hopkins, Stanford University, Harvard University, Brown University, University of Puerto Rico, University of Guam, King's College London, University of Northern British Columbia, and Murdoch Children's Research Institute in Australia. In addition, participants came from 51 different academic departments demonstrating the multi-disciplinary reach of the training. The nine departments with the most participants represented can be found in Table 1. Public Health (28.4%), Tropical Medicine, Medical Microbiology, and Pharmacology (7.8%), and Native Hawaiian Health (5.0%) were the top three departments represented in the sample. Other departments not shown in Table 1 include Chemistry, Genomics, Anthropology, Psychiatry, Mathematics, and Kinesiology.

**Figure 1:**
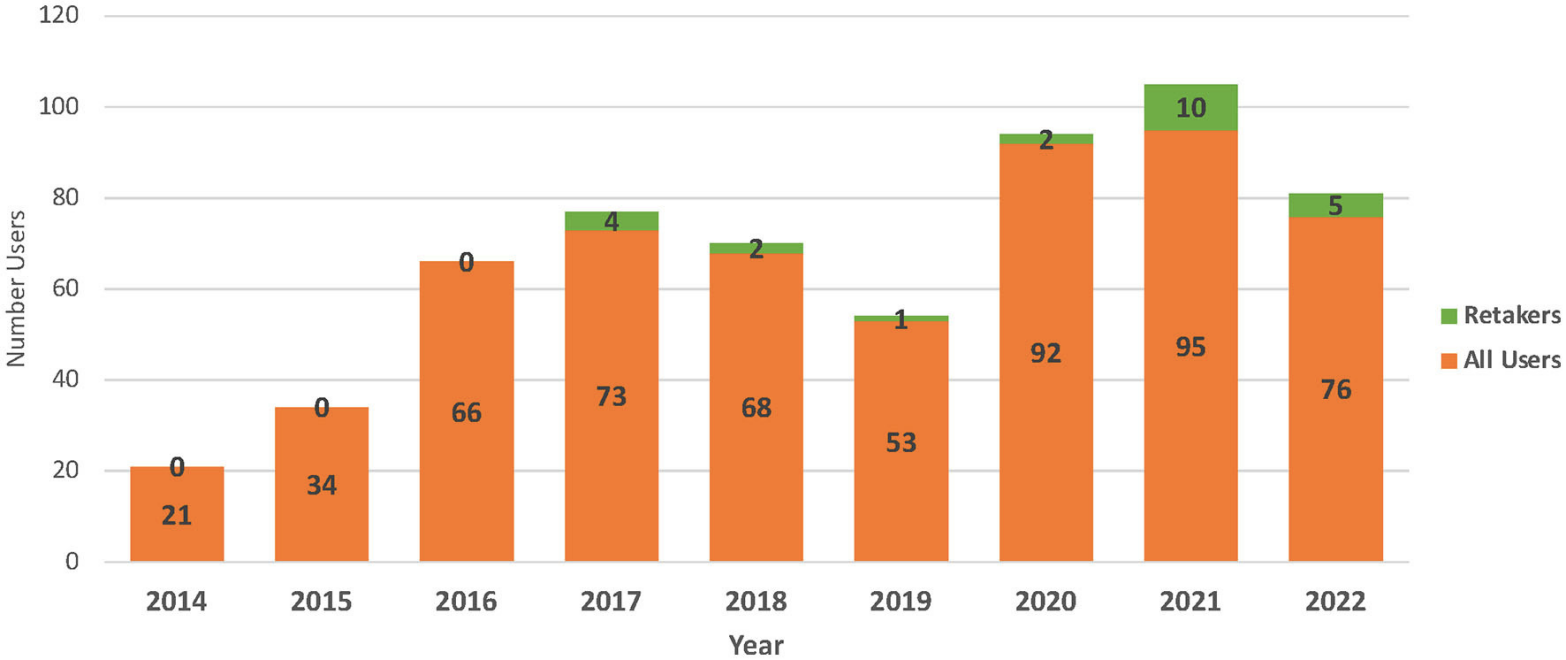
Number of users completing community 101 program by year (*N* = 602).

Participants were asked to briefly described their interest in completing the training. A total of 352 participants (50.5%) provided an answer with many indicating that the program had been assigned as a requirement for an academic course or their employment. Courses mentioned were those in university departments, such as public health, and training requirements included research internships and fellowships for undergraduate and graduate students. Participants also reported their personal and professional interest in engaging in community-based participatory research with underserved communities such as Native Hawaiians, immigrants, and Pacific Islanders. In addition, they described a desire to be collaborative with community members in research and wanting to relate and be prepared to work with the people of Hawai‘i. In examining the quizzes, a total of 602 non-duplicative users completed the Community 101 Program over the course of nearly 9 years (126 months) (Figure 1). On average, nearly 70 users completed the Community 101 Program each year with few multi-year users (mean = 2.7 users/year, range 0–10).

### 3.2 Optional curriculum evaluation and suggestions

**Figure 2:**
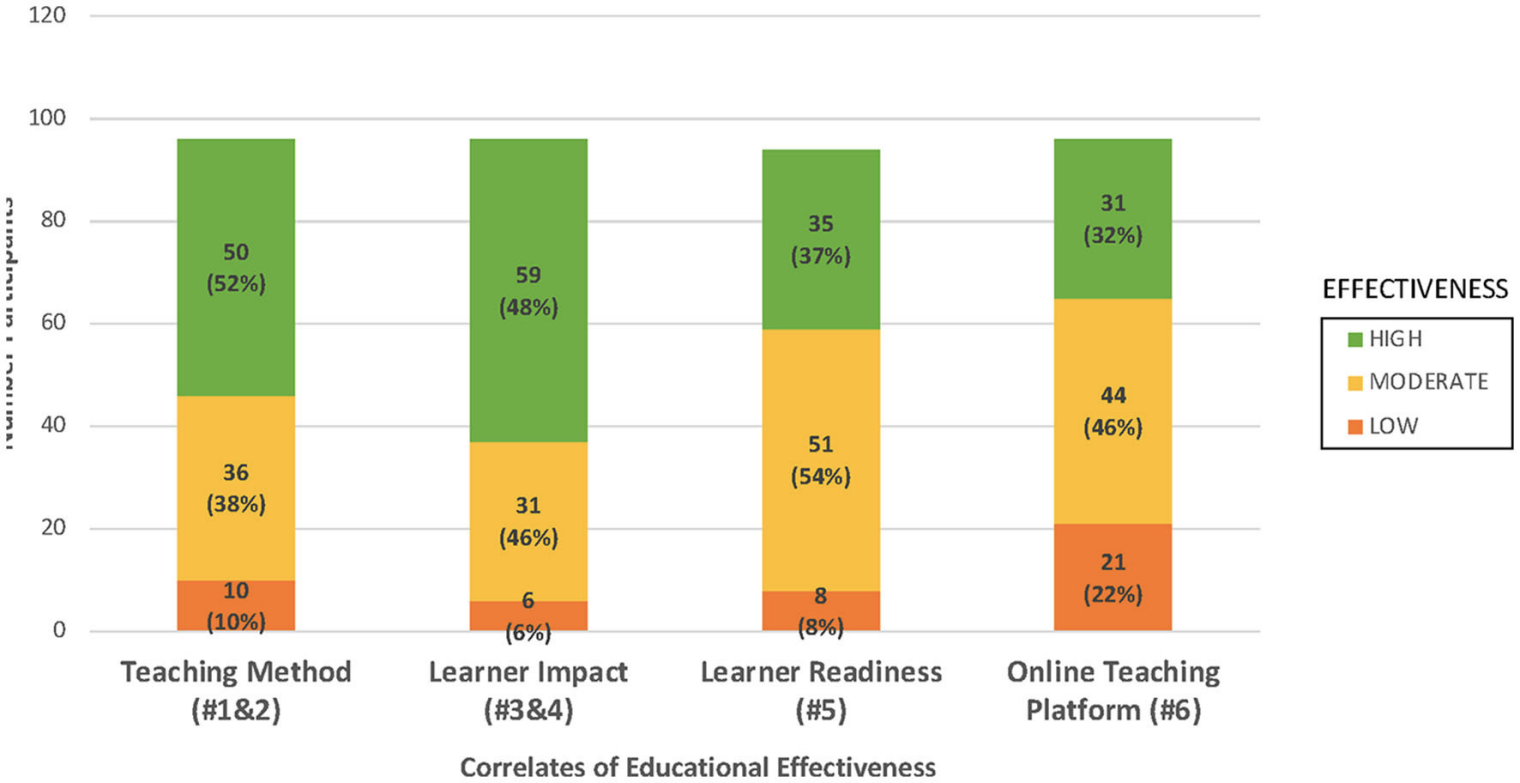
Evaluation of community 101 program for educational effectiveness (*n* = 96).

A total of 96 participants completed an optional evaluation survey (six survey items) that measured their perception of the training program. See Figure 2 for the results. The majority of participants (90% and higher) strongly agreed or agreed that the overall teaching effectiveness of the instructors and the overall quality of the modules was excellent, the presentations were clear and easy to understand, they learned a great deal from these presentations, and they feel well-prepared to interact with communities in developing research projects. Approximately 78% of participants strongly agreed or agreed that the software program used to present the modules was excellent. Out of the 96 participants who completed the optional evaluation survey, 58 qualitative responses were given to the question, “What are the major strengths of the modules?” The common strengths identified are listed in Table 2. Participants generally found the training to be clear, organized, and engaging with good pace and length. The topics covered, especially their relevance to and real-life examples from the Native Hawaiian and Pacific Islander communities, were also commended. As one participant stated. “*I think each topic was covered in a way that was easy to understand. The utilization of real projects was enticing to understand that these methods work.”* For the question that asked, “What do you suggest to improve the modules?” a total of 53 participants responded. Suggestions focused mostly on technical issues such as volume, as well as more content related suggestions such as more interactive, specific, and updated content. Some participants asked for transcripts of the audio. As one participant stated, “*providing a transcript would be nice so that we could read at our own pace, plus it would allow us to review information we were interested in more easily.”*

**Table 2:** Common qualitative themes from respondents on an optional evaluation survey of the community 101 for researchers training program (*n* = 96).

**What are the major strengths of the modules? (*n* = 58)**	**What do you suggest to improve the modules? (*n* = 53)**
•Clear and organized slides. •Engaging visuals and audio. •Good pace and length. •Perspectives from both community and experienced academics, especially the community interviews. •Real life local examples that are culturally tailored. •Cultural-historical background of the NHPI communities.	•Improve audio quality to address low or fluctuating volume. •Grammar and visual checks to correct words that are cut off words or typos. •Provide written transcripts to improve understanding of spoken words (i.e., closed captions). •Update the content periodically. •More interactive component. •Add examples of specific techniques to approach communities.

## 4 Discussion

Over the years, the “Community 101 for Researchers” program has become required for many students enrolled in public health courses, summer research internships at the John A. Burns School of Medicine, local hospitals, and highly recommended for community-based organizations working with academic partners or other research groups. Although the majority of the users were based in Hawai‘i, the Community 101 training program was accessed from respondents in other US states and territories and countries outside of the US, suggesting a broad global distribution of individuals who may be interested in learning about ethically conducting research with historically marginalized populations. In addition, the number of users over the years since its launch in 2014 has been steady and in fact, actually increased during 2020 to 2022 compared with the first 3 years (2014–2016). The exact reason for this increase is not known but should be explored in the future to better understand the demand and needs for these types of trainings. Given that we have not made any systematic efforts to publicly advertise this training program aside from posting the link on the RMATRIX website suggests that this approach may be useful in other settings in which researchers may be interested in learning how to ethically and effectively engage with diverse populations. Both the quantitative and qualitative evaluation feedback from participants has largely been positive with most commending the culturally relevant examples and stories from the community.

It is important to note that the limitations of the Community 101 program and this study. Given the pragmatic intent to focus specifically on Native Hawaiian and Pacific Islander communities, the training may not be generalizable to other populations. Moreover, this evaluative analysis did not assess how and if the participants intended to apply the knowledge from the training to their research. To evaluate the long-term effectiveness of this training, more studies need to be done to assess how learners are incorporating the content of these modules in their actual applied work. We plan to collect follow-up data to assess the effectiveness and long-term outcomes of this training. For example, demographics factors of researchers may be assessed in the future to determine if they play a role in their abilities to effectively apply the content to their real-life engagement with communities. Qualitative studies may also help us gather more information on real-life examples of research conducted and their challenges and successes after completing the Community 101 Training. Nevertheless the current study helped us identify areas of improvement that can inform the future direction of the Community 101 Training Program, which include enhancing the technical aspects as well as updating the examples.

This training program is just one of many ways we can advance ethical community research and begin to share specific community perspectives and world views. Researchers should not only avoid community harm, but also actively ensure that communities truly benefit from research. To reach this goal, multi-pronged strategies must be implemented. For example, we should build the capacity of community members, not just academic researchers, to engage in research so communities have the knowledge and tools to ask the right questions and hold expectations for ethical conduct. The absence of culturally relevant research ethics curricula especially for marginalized and Indigenous communities can be significant barriers for communities to truly engage in research (37). For example, American Indian and Alaskan Native communities raised concerns about the CITI and NIH trainings being too lengthy, including jargon, lacking cultural and contextual relevance, and failing to recognize community risks and benefits (38, 39). To address this, Pearson et al. (40) co-developed and co-tested a culturally tailored training curriculum on research ethics and protocols to increase the engagement of American Indian and Alaskan Native community members as co-researchers and participants in research, which demonstrated higher total knowledge scores, higher levels of trust in research, and higher levels of self-efficacy (40). This finding speaks to the importance of culturally tailoring curricula that enhance research capacity for both community members as well as academic researchers.

We initially designed the Community 101 Training Program in response to multiple requests by academic researchers who were unfamiliar with Native Hawaiian and Pacific Islander communities but wanted to pursue research with such communities and thus, sought out the assistance of Native Hawaiian and Pacific Islander researchers to assist them in gaining access. Frequent and repeated requests for community access and community perspectives often place a significant burden on community-engaged researchers, who are often from communities of color (41). The additional expectations and burdens to share knowledge and provide access to communities can impede the trust and relationships built. Therefore, funding to develop these types of curricula that are widely accessible can help alleviate this burden. These programs can reach a wider audience in a more efficient manner. However, it is also important to note that the Community 101 Training Program helps to build an introductory foundation but should not be seen as a comprehensive tool that will fully prepare all researchers to engage in the complexities and nuances of engaging with communities to do ethical and meaningful research. Each community is unique and researchers must take the time to show up, listen, be of service, and assess IF there is a space or a role for them (27). Researchers who wish to pursue research with Native Hawaiian and Pacific Islanders must also be prepared to commit to the long-term relationships, which is one of the main principles of community-based participatory research.

In addition, educating researchers is not sufficient alone. Other institutional and structural changes need to occur to truly support ethical community-engaged research. For example, many tenure and promotion criteria award number of publications despite the fact that peer-reviewed journals are inaccessible to communities and not written in a way that is conducive to community impact (42, 43). Tenure and promotion guidelines should integrate community impact to encourage researchers to engage in the often slow process of building trust and listening carefully and thoughtfully to communities, including rewarding community co-authorships and community-friendly products. In addition, funding mechanisms should better promote power-sharing between academia and communities and provide support for the critical relationship-building that is needed for ethical community-engaged research (44). The University of Hawai‘i, with the goal to become a Native Hawaiian Place of Learning (45), and other universities in Hawai‘i by the Pacific are committed to and currently working toward more robust community engagement, ethical research, and utilizing Indigenous models of research. The University of Hawai‘i JABSOM, Chaminade University, and Hawai‘i Pacific University have recently been awarded a 5 year NIH-NIGMS Pacific Center for Innovations, Knowledge and Opportunities (PIKO) Health Equity Institutional Development Award (IdeA) for Clinical Translational Research capacity/infrastructure building, with a large community engagement core, which partners with Native Hawaiians, Pacific Islanders, Filipinos and other disenfranchised communities in Hawai‘i (46). The UH Cancer Center in partnership with University of Guam has an NIH-NCI Pacific Island Partnership for Cancer Health Equity (PIPCHE). This grant is in its third 5-year cycle, also with a significantly large community engagement core, to build cancer infrastructure/capacity with Native Hawaiians, Pacific Islanders and other underserved populations in Hawai‘i and the larger Pacific.

There are several limitations to this study. For example, no pre and post outcomes were measured. The evaluation survey was anonymous so we are unable to assess if those who answered the optional evaluation survey differ from those who did not complete the survey. In addition, we have not validated the questions on the evaluation survey. More questions that are tailored to the learning objectives could be added. Future studies include a follow up study with participants to assess if they retained what they learned and how/if they applied the concepts from the Community 101 Program to their work with communities. To strengthen the curriculum, we plan to update the content, possibly change the delivery mechanism to a more “social media” approach (e.g., YouTube), and include transcripts of the program to enhance accessibility. To enhance the potential reach of the training, the title of the training program could be updated to make the topical emphasis clearer to potential audiences. Although initially intended for researchers based in Hawai‘i, the findings demonstrate its wider utility and relevance. Therefore, advertisement and outreach efforts can be enhanced once the curriculum is updated and strengthened based on participant feedback. Because this paper describes the formative development and preliminary results of the evaluation of this training, the next steps would be to conduct a more complex mixed-method study to generate more information about program effectiveness and application. By continuing to strengthen various tools and approaches to advance ethical community-engaged research, health equity can be realized.

## Data availability statement

The raw data supporting the conclusions of this article will be made available by the authors, without undue reservation.

## Ethics statement

Ethical approval was not required for the study involving humans in accordance with the local legislation and institutional requirements. Written informed consent to participate in this study was not required from the participants or the participants' legal guardians/next of kin in accordance with the national legislation and the institutional requirements.

## Author contributions

JC-D lead writer, co-developed intervention, conceptualized the study design, and conducted data analysis. SS wrote sections of paper and conducted data analysis. BJ assisted with the technical aspects of the intervention co-production, extracted and conducted data analysis, and edited the paper. ML, DT, NP, and KF co-developed intervention program and edited paper. MM conceived of, co-developed, and co-produced the intervention program, conceptualized the study design, conducted data analysis, and edited paper. All authors contributed to the article and approved the submitted version.
